# WhatsApp and Gynecologist-Patient Interaction: Development and Validation of a Questionnaire to Assess the Stress Perceived by the Doctor

**DOI:** 10.1055/s-0042-1744289

**Published:** 2022-04-20

**Authors:** Melissa Gonzalez Veiga, Rogério Tadeu Felizi, César Eduardo Fernandes, Emerson Oliveira

**Affiliations:** 1 Faculdade de Medicina do ABC, Santo André, SP, Brazil

**Keywords:** mobile applications, occupational stress, physician-patient relations, smartphone, questionnaire, aplicativos móveis, estresse ocupacional, relações médico-paciente, smartphone, questionário

## Abstract

**Objective** Construction and validation of the WhatsApp Stress Scale (WASS), a questionnaire designed for physicians that measures how the use of smartphones and related software communication applications affects the quality of life of gynecologists who use this tool to communicate with patients.

**Methods** The present cross-sectional observational study analyzed 60 gynecologists according to weekly WhatsApp usage time for communication with patients and compared the data with the perception of the doctor on the use of this virtual interaction as a stressor. Physicians were equally divided into three groups: < 2 hours, 2 to 5 hours, and > 5 hours. The authors created a questionnaire in Likert scale format. The study proceeded in three phases: development of the questionnaire items, pretesting, constructing, and validity and reliability testing using factor analysis, Cronbach α coefficient, and paired *t* -test.

**Results** A 9-item instrument using a 5-point Likert scale was created and administered to the participants in 3 different times: T0, T1 (15 minutes after the end of T0), and T2 (15 days later). All questionnaire items possessed adequate content validity indices and the internal consistency of the instrument was satisfactory (Cronbach α 0.935; 95% confidence interval [CI]: 0.744–0.989; *p* = 0.0001). No statistically significant differences were observed in the responses between the rounds of testing, indicating good test-retest reliability. A positive association between the high frequency of WhatsApp usage for communication with patients and the stress perceived by the doctor was shown.

**Conclusion** The WASS is a valid and reliable instrument for assessing the use of messaging applications to communicate with patients as a stressor perceived by gynecologists.

## Resumo

**Objetivo** Construção e validação do WhatsApp Stress Scale (WASS, na sigla em inglês), questionário desenvolvido para médicos que avalia como o uso do smartphones e aplicativos de comunicação afetam a qualidade de vida dos ginecologistas que usam estas ferramentas para comunicação com pacientes.

**Métodos** O presente estudo transversal observacional analisou 60 ginecologistas de acordo com o tempo de uso semanal do WhatsApp para comunicação com pacientes e comparou os dados de percepção dos médicos do uso desta interação virtual como agente estressor. Os profissionais foram igualmente divididos em 3 grupos: < 2 horas, de 2 a 5 horas e > 5 horas. Foi criado um questionário no formato de escala tipo Likert. O estudo procedeu em três fases: desenvolvimento dos itens do questionário, pré-teste, construção, validação de constructo e teste de confiabilidade usando análise fatorial, coeficiente alfa de Cronbach e teste t pareado.

**Resultados** Um instrumento com 9 itens foi criado e administrado aos participantes em 3 tempos diferentes: T0, T1 (15 minutos após o término de T0) e T2 (15 dias depois). Todos os itens possuíam validade de conteúdo adequada e a consistência interna do instrumento foi satisfatória (alfa de Cronbach 0,935; intervalo de confiança [IC] 95%: 0,744–0,989; *p* = 0,0001). Não foi observada diferença estatisticamente significativa entre as rodadas de teste, indicando boa confiabilidade teste-reteste. Foi demonstrada uma associação positiva entre maior tempo de uso do WhatsApp para comunicação com pacientes e estresse percebido pelos médicos.

**Conclusão** O WASS demonstra ser um instrumento válido e confiável para avaliar o uso de aplicativos de mensagens para comunicação médico-paciente como agente estressor percebido pelo ginecologista.

## Introduction

With the advent of the Internet, the speed of information exchange and the introduction of newer communication media have dramatically improved the healthcare sector. 1 The emergence of social media has shifted information-seeking behavior in society and the health sector is not immune from this influence. 2 The digital revolution had a profound impact on how physicians interact with patients and the community and the increased use of smartphones and related software applications has created a new era in the exchange of clinical data between patients and clinicians. 3 Recently, low-cost instant messaging services (WhatsApp Messenger [WhatsApp Inc, Menlo Park, CA, US], Skype [Skype Technologies, Luxembourg City, Luxembourg] Telegram [Telegram Messenger Inc., London, UK], etc.) have practically replaced all electronic media used before thanks to the wide availability of smartphones worldwide. 4

Among innumerable communication applications for smartphones, WhatsApp is considered to be the most widespread and has been downloaded in 40 countries in Europe, Asia, the Middle East, and the Americas. 5 A growing number of healthcare workers have adopted WhatsApp in their daily work to share information with patients. 5 The app can be downloaded freely via the Internet and is available for all commonly used mobile platforms such as Android, iPhone, and Windows mobile. 6 Other advantages of using WhatsApp in healthcare are improvement of communication, no requirement for a computer, time saving, and the possibility of an immediate response. 3 7 8 9 Despite the many benefits, existing risks or disadvantages have also been reported: increase in workload by staying online 24 hours a day, disparity in the sense of urgency, worsening of professional relationships and risk of unprofessional behavior, clinical information not being included in medical records, possible issues of privacy and data protection, ethical aspects of clinical evaluations at a distance, and lack of specific legislation. 9 10 11 12 Besides, some disadvantages of using WhatssApp may be the error in the interpretation by the professionals of complaints of the patients and failures in the understanding of the recommendations by the patients. 4

It is not unusual for the demand placed on people by the changes in modern life and the consequent need to adjust to these changes to end up inducing a situation of conflict, anxiety, anguish, and emotional destabilization. 13 Stress ends up as a direct consequence of persistent efforts to adapt to the existential situation. 13 The association between smartphone use and increased stress was suggested by many studies. 10 14 Referred to as “communication overload,” Thomée et al. 15 found that this pressure to be constantly available was associated with higher smartphone use – to meet the expectation – and that this pressure was associated with feelings of guilt, stress, and depression.

Psychological distress, such as depression and anxiety, has become a major workplace mental health problem and could be associated with several symptoms and possible consequences among various professions. 16 For instance, depression could result in low productivity, absenteeism, job turnover, and economic costs, whereas anxiety, when it becomes excessive and persistent, is frequently accompanied by physiological symptoms such as headache, sweating, fatigue, or exhaustion. 16 17 Additionally, psychological distress among healthcare workers impairs not only their own health, but also imperils the health and safety of their patients. 18 However, to date, little research has examined the actual psychological experience of technostress itself. Thus, the present study aimed to present the construction and validation of the WhatsApp Stress Scale (WASS), a questionnaire designed for physicians that measures how WhatssApp use affects the wellbeing and quality of life of gynecologists who routinely use this tool as a means of communicating with patients.

## Methods

We conducted a cross-sectional observational study between August 2019 and July 2020 that was previously submitted to and approved by the Research Ethics Committee of the Faculdade de Medicina do ABC (FMABC, in the Portuguese acronym) under number 3.528.229. The population sample consisted of 60 gynecologists selected by convenience; the participants were actively recruited through the research of the own database of the group and were randomly invited. The inclusion criteria were as follows: agreement to participate in the study according to the informed consent form and gynecologists working in the state of São Paulo, Brazil, who use WhatsApp as a means of communication with patients. The authors developed the questionnaire after a thorough literature review and the items were generated based on the currently available literature and their basic research issue. 3 11 12 The questionnaire included general demographics and nine questions that examined the perception of doctors on the use of this virtual interaction as a stressor, according to the weekly WhatsApp usage time for communication with patients. The items were measured by a Likert scale ranging from 1 to 5 points (1, never; 2, almost never; 3, sometimes; 4, frequently; and 5, very frequently), and 9 items with scores ranging from 9 to 45 (the higher the score, the grater the perception of the use of the virtual communication with patients as a stressor). The 9-item list of the questionnaire is shown in Chart 1 .

**Chart 1:** Questionnaire items

How often did you answer messages unrelated to urgent matters? How often did you feel irritated by the messages? How often did you feel nervous about being asked out of working hours? How often did you feel uncomfortable with the lack of limits of the patient? How often did you notice a decrease in patience when having to reply to messages? How often did you feel insecure about this type of communication that does not have specific regulations or legal support? How often were you bothered by believing that this type of communication trivializes the medical service? How often did you feel irritated at not being paid to work through these tools? How often did you realize that being available to work with these tools interfered with your quality of life?

After the participants signed the informed consent form, demographic data were collected, such as age, gender, marital status, parity, physical activity habits, and graduate year. Next, the physicians answered the WASS questionnaire in a self-administered manner. The instrument was applied at 3 different times: the first, defined as T0, overseen by researcher A; the second, called T1, occurred 15 minutes after the end of T0, and was overseen by researcher B; and the third, 15 days later, called T2, when the instrument was completed via WhatsApp after being sent by researcher A. Regarding the psychometric properties to measure the questionnaires, the variables analyzed were internal consistency, test-retest reliability, and interobserver reliability. Internal consistency evaluates the correlation between the items and is determined from the subscale scores and the total score. A higher value indicates greater correlation between various items of the scale. A retest was performed 15 days after the first application of the questionnaire. The interobserver reliability was assessed by applying the instrument 15 minutes after the first interview. Finally, the discriminant validity was evaluated by applying the questionnaire in the three groups studied. The data were tabulated in Google Drive spreadsheets (Google LLC, Mountain View, CA, US). The software Prism version 8.4.1 (GraphPad Software, San Diego, CA, US) and IBM SPSS Statistics for Windows (IBM Corp., Armonk, NY, USA) were used for analysis. The normality of the data was analyzed by the Kolmogorov-Smirnov test and, as appropriate, analysis of variance (ANOVA), the Kruskal-Wallis test, and the Dunn test were used to compare continuous variables. The chi-squared test was used to compare categorical variables. The sample size was not calculated due to the extensive variability of formulas suggested to generate the minimum number of subjects for studies involving the validation of questionnaires. 19 The internal consistency of the instrument was calculated in the form of Cronbach α coefficient by measuring the 9 questionnaires items (> 0.9: excellent; 0.7–0.9: acceptable to good; 0.6–0.7: questionable; 0.5–0.6: poor; and < 0.5: unacceptable). 20 The test-retest reliability was calculated with the intraclass correlation coefficient (ICC), which allows us to determine whether the studied tool is reliable for comparing the scores obtained at T0 and T2 (intraobserver) and at T0 and T1 (interobserver). When ICC = 0, the questionnaire is not reproducible; when ICC = 1, the questionnaire has maximum reproducibility; an ICC < 0.4 means that the reproducibility is poor; an ICC ≥ 0.75, excellent reproducibility; and for 0.4 ≤ ICC < 0.75 the reproducibility is satisfactory. 21 All statistical tests were two-tailed, with a significance level of 5%.

## Results

We included 60 gynecologists who were equally divided into 3 research groups according to the weekly WhatsApp usage time for communication with patients: ≤ 2 hours (GI), 2 to 5 hours (GII), and > 5 hours (GIII). Participants in GII had a higher mean age than those in GI and GIII (46.7 ± 9.5 versus 44.2 ± 9.5 and 41.8 ± 6.8 years old, respectively; *p* = 0.23). GII also had a greater proportion of individuals who were married (70%; *p* = 0.75) and had at least 1 child (80%; *p* = 0.16). More than half of the doctors self-identified as a female in all groups ( *p* = 0.76). The prevalence of physical activity was higher in GIII ( *p* = 0.41). Most participants in GI, GII and GIII had been working for > 15 years as a doctor (75, 85, and 65%, respectively; *p* = 0.34) and had a weekly workload of > 30 hours (90, 80, and 90%, respectively; *p* = 0.56). Table 1 shows the demographic characteristics of the study population in more detail.

**Table 1:** Demographic characteristics

Variable	< 2 hours ( *n* = 20)	2–5 hours ( *n* = 20)	> 5 hours ( *n* = 20)	*p-value*
Age a	44.2 ± 9.5	46.7 ± 9.5	41.8 ± 6.8	0.23
Gender b				
Female	16 (80)	14 (70)	15 (75)	0.76
Male	4 (20)	6 (30)	5 (25)	
Marital Status b				
Married	12 (60)	14 (70)	12 (60)	0.75
Single	8 (40)	6 (30)	8 (40)	
Child b				
Yes	11 (55)	16 (80)	11 (55)	0.16
No	9 (45)	4 (20)	9 (45)	
Physical Activity b				
Yes	9 (45)	8 (40)	12 (60)	0.41
No	11 (55)	12 (60)	8 (40)	
Graduate Year b				
≤ 15 years	5 (25)	3 (15)	7 (35)	0.34
> 15 years	15 (75)	17 (85)	13 (65)	
Weekly Workload b				
≤ 30 hours	2 (10)	4 (20)	2 (10)	0.56
> 30 hours	18 (90)	16 (80)	18 (90)	

a Values presented as mean and standard deviation. Statistical test: analysis of variance (ANOVA).

b Values presented as numbers and percentages. Statistical test: chi-squared test.

Doctors who reported spending more hours a week communicating with patients via WhatsApp had higher levels of stress ( *p* = 0.0024). Highest stress scores did not correlate with gender, marital status, having a child or graduate year. However, there was a significant association between age and perceived stress. Younger physicians had higher stress scores. Table 2 shows the analysis of the WASS scores in the three groups studied. Discriminant validity was demonstrated.

**Table 2:** Discriminant validity of the WhatsApp Stress Scale questionnaire between groups

< 2 hours	2–5 hours	> 5 hours	*p-value*
32 (15–45)	34 (16–45)	35.5 (25–45)	0.0024

a Values presented as median, minimum and maximum, range. Statistical test: Kruskal-Wallis and Dunn multiple comparison test. Multiple comparisons: GIII > GII ( *p* = 0.0457) and GIII > GI ( *p* = 0.0023).

The internal consistency values of the WASS questionnaire were found for all items, and for the overall score, the Cronbach coefficient was 0.935 (95% confidence interval [CI]: 0.744–0.989; *p* = 0.0001), showing excellent internal consistency. Therefore, the reliability of the instrument was adequate. Finally, no differences were observed in the test–retest comparison of the WASS questionnaire in the intra- or interobserver evaluation. The ICC results were 0.838 and 0.847, respectively, showing excellent reproducibility (ICC > 0.75) ( Table 3 ).

**Table 3:** Test-retest intraobserver and interobserver reliability of the WhatsApp Stress Scale

Intraobserver ICC; 95%CI; *p-value*	Interobserver ICC; 95%CI; *p-value*
0.838; 0.731–0.903; 0.0001	0.847; 0.742–0.909; 0.0001

Abbreviations: CI, confidence interval; ICC, intraclass correlation coefficient.

## Discussion

The advent of telemedicine has allowed physicians to deliver medical treatment to patients from a distance. Mobile apps such as WhatsApp Messenger came as a novel concept in all fields of social life, including medicine, offering and disseminating scientific and technological information. 5 22 Because of the quick development and widespread use of mobile phones and of their vast effect on communication and interactions in work and private life, it is important to study possible negative health effects of exposure to them. 14 However, in different countries of the world, few investigations have been conducted on the attitudes and opinions of physicians about the use of social media in the healthcare field. 22 The present study aimed to develop and validate a questionnaire to assess the effects of WhatsApp use on the wellbeing and quality of life of gynecologists who routinely use this tool as a means of communicating with patients.

The widespread use of technology and the electronic environment in the healthcare system and its effect on physicians have been suggested by some prior research, but according to our knowledge, this is the first study to date to look at WhatsApp as a stressor for physicians in the context of the doctor-patient relationship. 23 The process of developing the WASS resulted in a 9-item questionnaire. The face validity was satisfactory, and a good discriminant validity was shown. The instrument also showed good internal and external reliability, demonstrating that the questionnaire is measuring the wellbeing and quality of life of gynecologists who use WhatsApp to communicate with patients consistently and reproducibly.

We found that the highest stress scores among gynecologists were associated with more hours spent communicating with patients via WhatsApp. Similarly, studies have shown prospective associations between instant messaging and perceived stress. 24 25 Considering the variables examined, there appeared to be a significant difference of stress scores across age groups. The present study found that younger respondents demonstrated the highest levels of stress, which is in line with the findings of Ragu-Nathan et al. 26 that technostress decreased as age increased. The assessment of workload was based on self- reporting; thus, it is possible that those professionals reporting higher levels of stress perceived that they worked more hours. Therefore, the effect of age on the perceived stress warrants further investigation.

In terms of gender, male and female gynecologists did not differ significantly in their perceived stress, contrary to previous research that found clear associations for women, in whom chatting online was associated with prolonged stress and symptoms of depression. 25 Our results did not correspond with those of previously conducted studies that concluded that the prevalence of physical activity may have some impact on improving mental health and quality of life. 27 There were no significant differences in perceived stress related to marital status, having children or graduate year. Panagopoulou et al. 28 found that work hours are not a predictor of stress, which is in line with our results, which show that the raw objective data may not be the only appropriate variable to correlate with stress.

More and more studies have linked the overuse of the Internet and of smartphones to lower life satisfaction and higher stress. 29 30 31 Stress poses a substantial problem for the wellbeing of physicians and for the quality of healthcare and is likely to be harmful to relationships with patients. There is ample evidence that doctors who are under high levels of stress deliver poorer patient care and make potentially critical mistakes. 28 32 Identifying the risk factors of stress may prevent medical errors from occurring and increase the satisfaction with health care. Our findings serve as a reminder that this relationship between the use of smartphones to communicate with patients and stress exists, allowing gynecologists to be aware of this association in their daily lives.

There were some limitations in the development of the present questionnaire. The sample size of the study is relatively small; a larger sample could have highlighted missing aspects. Besides, the instrument was applied in specialists in the same area of expertise, which could limit the generalizability of the study. In addition, the construct validity of the instrument was not assessed, as correlations between our scale and existing, validated assessment tools of quality of life were not established.

## Conclusion

The present questionnaire was found to be a valid and reliable instrument with a high internal consistency for the evaluation of WhatsApp usage in the patient-doctor interaction as a stressor for gynecologists who routinely use this tool to communicate with patients.
